# Being Present in Action: A Theoretical Model About the “Interlocking” Between Intentions and Environmental Affordances

**DOI:** 10.3389/fpsyg.2015.02052

**Published:** 2016-01-22

**Authors:** Stefano Triberti, Giuseppe Riva

**Affiliations:** ^1^Department of Psychology, Università Cattolica del Sacro CuoreMilan, Italy; ^2^Applied Technology for Neuro-Psychology Lab, Istituto Auxologico ItalianoMilan, Italy

**Keywords:** intentions, presence, action, agency, affordance

## Abstract

Recent neuropsychological evidence suggest that a key role in linking perceptions and intentions is played by sense of presence. Despite this phenomenon having been studied primarily in the field of virtual reality (conceived as the illusion of being in the virtual space), recent research highlighted that it is a fundamental feature of everyday experience. Specifically, the function of presence as a cognitive process is to locate the Self in a physical space or situation, based on the perceived possibility to act in it; so, the variations in sense of presence allow one to continuously adapt his own action to the external environment. Indeed intentions, as the cognitive antecedents of action, are not static representations of the desired outcomes, but dynamic processes able to adjust their own representational content according to the opportunities/restrictions emerging in the environment. Focusing on the peculiar context of action mediated by interactive technologies, we here propose a theoretical model showing how each level of an intentional hierarchy (future-directed; present directed; and motor intentions) can “interlock” with environmental affordances in order to promote a continuous stream of action and activity.

## Introduction

Recently, Riva et al. (2011, 2015a), Riva and Mantovani (2012), Waterworth and Riva (2014) proposed that a fundamental role in coupling intentions, perception, and action is played by *sense of presence*, conceived as a specific cognitive process. The concept of sense of presence emerged around the Nineties in the field of interactive technology studies, in particular in Virtual Reality. Indeed, the first studies tried to understand what allowed people to feel present inside computer-simulated environments.

The so-called *Media Presence* theories (Loomis, 1992; Sheridan, 1992, 1994; Schloerb, 1995; Lombard and Ditton, 1997) consider sense of presence as the function of the experience of a given medium. These theories explain sense of presence on the basis of perception and attention. For example, according to Lombard and Ditton (1997) sense of presence appears when an “illusion of non-mediation” establishes, that is, the individual using virtual reality stops to pay attention to the technology in use (for example, the head mounted display) and focuses on the content of the virtual environment. On the one hand, these theories are useful to provide virtual reality design guidelines. On the other hand, these theories fail in explaining why something like the sense of presence exists. Why, from an evolutionary point of view, should we need something like a cognitive process devoted to generate a sense of “being there” while interacting with simulation technologies? *Media Presence* theories do not provide answers to this question (Lee, 2004; Riva et al., 2011). The existence of sense of presence highlights that the perceived location of an individual is not a mere by-product of the individual actually being in a given place. Indeed, since the manipulation of environmental features (for example: digitally rendering a simulated environment via virtual reality) is able to alter the perceived location of the individual, it appears that a specific form of information processing is devoted to provide such outcome. We can consider critically the proposal by Lombard and Ditton (1997): if sense of presence depends on an illusion excluding the virtual reality technology from our attention, how can we feel a sense of presence in physical reality, where no technological mediations exist?

In contrast, *Inner Presence* theories (Zahorik and Jenison, 1998; Moore et al., 2002; Lee, 2004; Riva et al., 2011, 2015b; Riva and Waterworth, 2014) consider sense of presence as a fundamental component of our cognition, which plays a precise role in our everyday life and is not necessarily related to the fruition of interactive media. Riva et al. (2011, 2015b) proposed a complex model that describes sense of presence as a neuropsychological phenomenon whose central goal is the control of agency and activity, through the unconscious separation of “internal” and “external”. In other words, the experience of presence can be described as the outcome of an intuitive meta-cognitive process that allows us to control our actions through the comparison between intentions and perceptions (Riva and Mantovani, 2012). Following this view, presence is a core neuropsychological phenomenon whose goal is to produce a sense of agency and control: I am present in a real or virtual space if I manage to put my intentions into action (enacting them). Feeling variations in the sense of presence, one can monitor his own actions and tune his activity accordingly.

According to this theory, the link between sense of presence and the enacting of intentions is strong and fundamental. If we consider again the field of virtual reality and new media, indeed, technical aspects of the virtual environments (such as, for example, pictorial realism or intensity of the sensory stimulation) have a weak impact on the sensation of “being there” if compared with the impression of being able to enact intentions. For example, a video game player can feel strongly present while playing a product with very simple graphics and basic animations. This could happen in that the game features:

(1) an usable and easy-to-master interface which is adequate to the player’s capable movements.
(2) an easily understandable game structure (possible directions to take, objectives, game rules, etc.) which efficiently relates to the here-and-now desired outcomes for the player.
(3) a compelling, engaging and interesting storyline, which provides the player with long-term goals and ultimate objectives.

Indeed, numerous experiments demonstrated that self-reported sense of presence in virtual environments is strongly related to the usability and effectiveness of interactive features (Coelho et al., 2006) and to narration/storyline contents (Gorini et al., 2011; Triberti et al., 2014). According to *Inner Presence* theories, this aspects influence sense of presence also in everyday life. One can feel more or less present in a given situation depending on how much he has the impression of being able to enact his own intentions, recognizing and using environmental opportunities for action, and then monitoring the perceived action outcomes as more or less consistent with the representational content of intentions. But how does this happen? What does it mean to “feel present” in everyday life? How does exactly sense of presence relates to one’s own Self?

**FIGURE 1:**
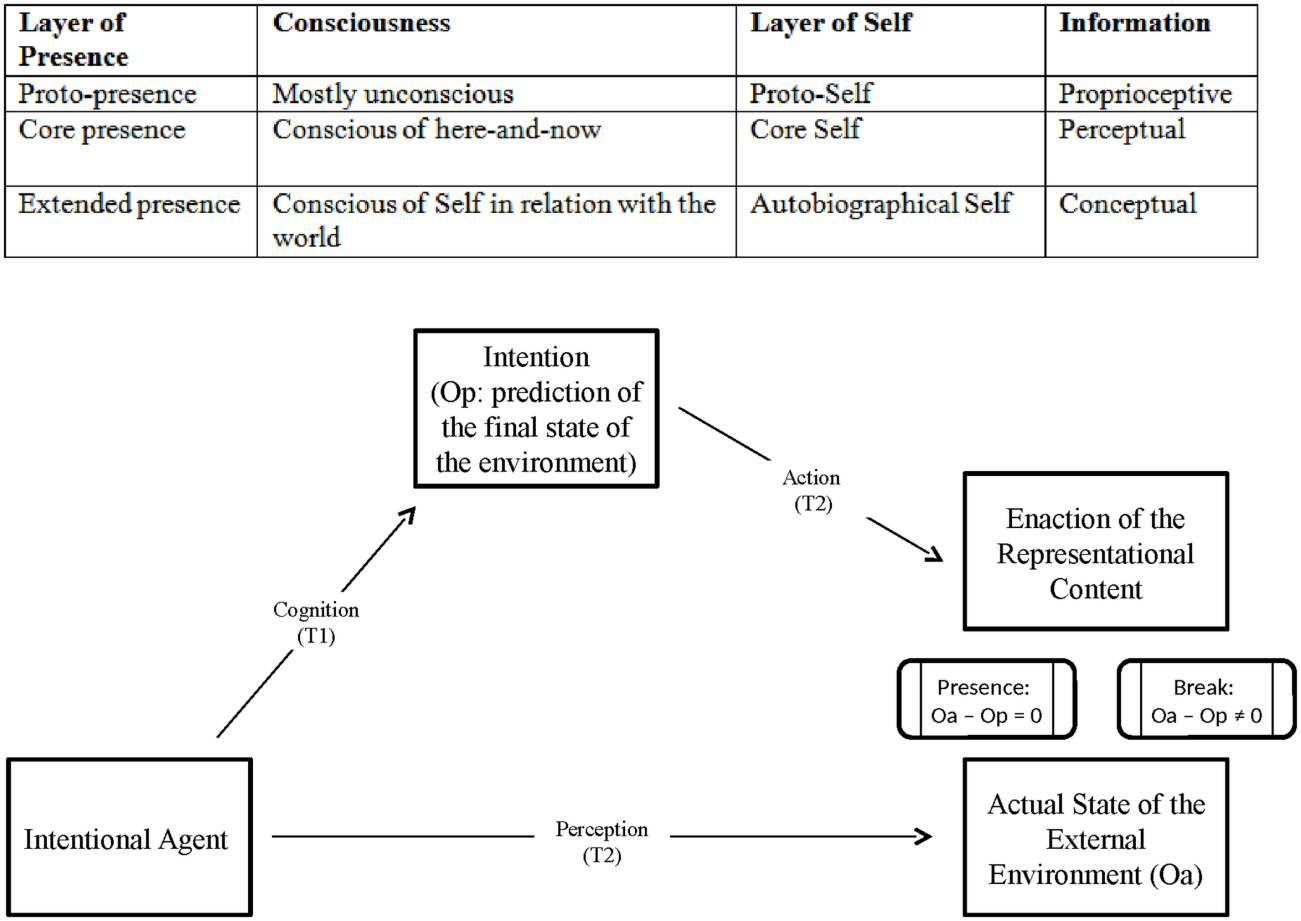
**Layers of presence and layers of Self, and a representation of the feeling of presence emerging from intention enacting and action monitoring (adapted from Waterworth and Riva, 2014)**.

According to Riva et al. (2011, 2015b), Waterworth and Riva (2014) sense of presence is a unitary feeling, but on the process side it can be divided into three phylogenetically different layers/subprocesses. On the side of Self, these layers are symmetrical to the layers of Self as described by Damasio (2011). According to him, the conscious Self is built, as a first step, on a collection of “primordial feelings” constituted by enteroceptive, proprioceptive and motor information coming from the body (proto-Self), which allow the organism to distinguish itself from the external environment. At the second level, core-Self is related to the perceptual differentiation between the Self and the recognized external object. Finally, the Autobiographical Self is related to the emergence of consciousness and symbolic/categorical knowledge: thanks to the use of language, we become able to represent the events in our personal story and, as a consequence, also to formulate abstract action plans oriented to the distant future. According to this layered conception of the Self, sense of presence can be represented as composed by three sub-processes. *Proto-presence* is the process of internal/external separation related to proprioception and motor control, since its object is the basic distinction between Self and non-Self, still without differentiating the characteristics of the external object(s). Basically, a sense of proto-presence allow us to monitor whether motor intentions are being correctly enacted by our own body, regardless of the external environment. Differently, *Core presence* is related to the sensorial experience of the environment. At this layer, the agent starts interacting with the objects. The “external” is specified at the level of affordances for actions, so, the agent monitor his own intentions as having or not the expected effects on the external environment which is around him at the present moment. Finally, *Extended presence* is to verify the significance to the Self of experienced events in the external world. The more the Self is present in significant experiences, the more it will be able to reach its goals, increasing the possibility of surviving. Extended presence requires intellectually and/or emotionally significant content. In other words, feeling extended presence means monitoring the enacting of abstract/general objectives into complex action plans. **Figure 1** shows how the three layers of presence relate to the Self as explained by Damasio (2011), and how intention enacting generates sense of presence through the confrontation between action and the final state of the external environment.

The theory of sense of presence highlights that we continuously monitor our own activity (in the form of intention enacting) in our own body, and in the external world. In this sense, intentions should be able to “interlock” with the opportunities for action coming from the environment, both at the level of the current situation and at the level of the extended, conceptual possibilities. On the one hand, this relates to activity monitoring when the action is initiated. For example, recent hypotheses coming from Neuroscience (Numan, 2015) sustain that the hippocampus and the prefrontal cortex play a critical role in the enacting of action plans and the formation of episodic, autonoetic memories; specifically, the prefrontal cortex is responsible for elaboration of goal-directed behavior and transmission of an efference copy of the action (or “corollary discharge”) to the hippocampus, which serves as an intention-outcome comparator. Finally, the response of the hippocampal comparator returns to the prefrontal cortex where it is used to strengthen the current action plan (in case of success) or to reformulate it (in case of intention-outcome mismatch), this way fostering memory updating. On the other hand, in the present contribution we will try to show how a comparison between intentions and the external world happens even before the action. The “interlocking” metaphor highlights that, as we will deepen in the next section, intentions should be conceived as layered structures themselves. Indeed, in order to an intention being enacted, any layer of an intentional structure (distal-conceptual; proximal-present; motor-micropresent) should find its own correspondence in the external world, in terms of feasible affordances. In this sense, “monitoring our own activity” does not mean to control for the consequences of actions only, but also, and more importantly, to perceive and recognize the affordances for action relying in the external environment before the action onset. How does this process actually happen? In order to explain this, we will deepen the concept of intention itself, showing how also intentions can be represented as hierarchies/layered structures; then, we will introduce a theoretical model about the interlocking between intentions and environmental affordances at the different levels of information processing.

## Intentions

Numerous philosophical conceptions, as well as common sense, posit that intentions guide actions. Classic experiments and theories challenged this apparently simple assumption, in that they have shown that neural activation related to the initiation of movement (readiness potential) seemed to appear independently of conscious awareness (Libet, 1999, 2010). For this reason, according to Libet (1999, 2010) and other researchers (Wegner, 2002), intentions may not be the causal antecedents of action, but an illusion generated by the consciousness after the action onset.

The paradox highlighted by Libet’s (1999, 2010) work can be resolved when considering a more sophisticated conception of intentions (Gallagher, 2006; Pacherie and Haggard, 2010). Indeed, intentions are not only motor representations guiding the motor components of an action and appearing just immediately before the movement itself, so that they can be fully associated with the readiness potential. On the contrary, they may develop at larger time scales (potentially, including almost a lifetime between the generation of the intention and its achievement) in that they entail conscious deliberative decisions, abstract, and descriptive representations, and “mental time travel” as a cognitive adaptation allowing humans to simulate contingencies and consequences of future actions (Suddendorf and Corballis, 2007; Eren, 2009; Corballis, 2013).

Recently, Pacherie (2006, 2008), Pacherie and Haggard (2010) introduced a dynamic theory of intentions which distinguishes among Distal Intentions, Proximal Intentions and Motor Intentions.

- Distal Intentions develop at potentially large time scales and represent abstract reasoning about means and plans (e.g.,: “I want to become a psychologist”)
- Proximal Intentions constitute the conscious antecedents of a given action, developing at the level of the present time (e.g.,: “Now I will write a psychology essay to pass my university exam”)
- Motor Intentions develop at a micro-present level and guide the motor components of the action (i.e., how the action should be physically performed); they are mostly unconscious (e.g.,: “I’m moving my hands on the keyboard *this way* to write”).

As Pacherie (2008) says, the three layers of intentions do not simply coexist, but they form an “intentional cascade” with Distal Intentions generating Proximal Intentions, and Proximal Intentions generating Motor Intentions. However, it is not fully clear how can the different layers of intentions relate to the external world. Indeed, Motor Intentions are strictly dependent on the physical environment where the movement is about to take place; for example, the motor intention “moving my hands on the keyboard *this way* to write” should “take into consideration” distance between the body and the computer, the position of the keys on the keyboard, as well as the strength to use with the fingers in order to correctly activate the keys.

Similarly, the intention “now I will write an essay”, as a proximal intention, has to “anchor the action plan in the current situation” (Pacherie, 2008, p. 188). Enacting a proximal intention means identifying the environmental affordances which permit the activation of the behavior. For example, the computer having a precise set of functions which allows one to write, cite and save his own work. In other words, at the level of proximal intentions, an agent should perceive and identify the opportunities for action existing within the environment. This happens not only at the mere motor level, but also identifying the functions of tools, the limits imposed by obstacles, the possibility to move or not to a different environment, and so on.

From the point of view of intention enacting, the Distal Intention (“I want to become a psychologist”) is the most elusive. Pacherie (2008, p. 188) sustains that distal intentions provide proximal intentions with an action plan that “may be still mostly descriptive and abstract”. Indeed, they are not directly related to the context of action (one may intend to become a psychologist independently of what is happening around him at the present moment). But how do distal intentions relate to the external world?

Castelfranchi (2014, p. 107), who is interested in showing that intentions are a specific kind of goals, define intentions as “those goals that actually drive our voluntary actions or are ready/prepared to drive them”. Also he focuses on trying to understand how abstract intentions can guide actions. He argues that abstract intentions need to be converted in “concrete cues”, so that it becomes possible for the agent to control whether they have been achieved or not. For example, a distal and abstract intention such as “I want to take revenge for the offense” should be situated in a precise situation (or multiple situations) in which precise actions (insulting, manipulating, dueling) take place. Doing so, an agent can effectively control his own intentions and agency. However, the concept of “concrete cues” appears as somewhat elusive. What are “concrete cues” exactly? On the one hand, they are probably effects and micro-effects of the action, that the agent compare with the representational content of the intention to monitor whether the action is being performed as desired/expected, as it is argued by the Comparator Models of agency (Pacherie, 2008; Carruthers, 2012; Chambon et al., 2014; Numan, 2015). However, this kind of “concrete cues” (i.e., those coming from the detection of action consequences) seem not sufficient to us as explanation of intentions. In the next section, we will introduce a theoretical model about the “interlocking” between intentions and environmental affordances, in order to show how intentions can relate to the external world even in absence of “concrete cues” conceived as consequences of performed action.

## Introducing a Model About the Hierarchical Interlocking of Enacted Intentions

We argue that an agent should not “control” an intention just at the time the intention is already in the form of its physical enaction. In other words, and agent should know whether a given intention can be enacted or not, already at the time when the intention is distant-future directed, abstract and merely descriptive, still not specified into physical actions and micro-movements at the motor level. Specifically, the agent should know whether his own intentions satisfy or not criteria different from the ones the agent himself uses to monitor effectiveness of physical action. At each level of the intentional hierarchy, intentions are the object of a cognitive/intuitive evaluation which authorizes them to proceed down the cascade until the initiation and the monitoring of behavior. But what are the concrete cues at the level of abstraction? How can we know whether a given cognitive process promoting behavior deserves the status of “intention” (e.g., goal guiding action or ready/prepared to guide it)? This kind of concrete cues allow the formation of an intention since they consist of information attesting intention’s “enactability” in the external world. As Laurent (2003) observes, mental representations (and also intentions) do not represent the state of the external world, but the state of one’s own engagement in the world. This means that intentions have to reflect the opportunities for enacting an action, this way becoming “simulated affordances” that illustrate what reality affords to enact behavior, at any level of information processing.

Indeed, each level of the intentional hierarchy is characterized by an external world-dependent requisite to be satisfied, in order to continue to guide action. So, we sustain that a given intentional hierarchy has to “interlock” with the external world, already at the time when the first movement(s) of the corresponding action are still not initiated.

- The motor intention is the intention that directly informs the movement. For this reason, it has to interlock with physical objects and properties which make possible, totally, or in part, the actual performance. Spatial and temporal limitations belong to this category, such as physical properties of objects that make them more or less adequate to the morphological structure of the agent. The condition for a cognitive process becoming a motor intention is having a representational content which is directly *tangible/manipulable*.
- The proximal intention consists in the product of the distal intention, as a general goal that is transformed into action plan(s). The proximal intention interlocks with environmental affordances, conceived as opportunities for action: it has to identify sets of functions, rules and provisions, in the form of possible courses of actions. For example, the intention “writing now an essay” requires the agent being able to recognize the environmental features which make possible the intention enacting, such as having the computer, turning it on and being able to write through the keyboard. To sum up, the condition for a cognitive process becoming a proximal intention is having a representational content which is *perceivable* as a concrete opportunity for action in the external world.
- The Distal Intention (“I want to become a psychologist”; “I want to take revenge of the offense”; “I want to win the gold medal”) interlocks with socio-cultural conditions which maintain a relationship of resemblance with the “possible world” imagined by the agent, that is, those contents of his imagination featuring emotional and identity values. For example: “I want to become a psychologist” is not an abstract claim; it actually develops in a socio-cultural context where (1) some desirable outcomes for the agent (e.g.,: understanding people; studying behavior; treating psychopathologies) are incarnated in the “psychologist” figure, and (2) that socio-cultural context features a number of possible courses of action one could take to actually become a psychologist. The condition for a cognitive process becoming a distal intention is to have a representational content which is *thinkable*, basing on the characteristics of the socio-cultural context in the external world.

Intentions (from the motor level to the more abstract one) are already interlocked with the world even before they are transformed into actions, because they are fundamentally predictive. The human mind has the capacity to generate probabilistic models about the future, basing on the analysis of sensory inputs. According to the so-called “free energy” framework (Friston et al., 2006; Friston, 2009, 2010; Fotopoulou, 2014), the fundamental function of our brain is to reduce the inconsistency between predictions about the world and the world as it is actually perceived, or, monitoring the divergence between our motivations/needs and the “state of the coupling between the individual and his environment” (Laurent, 2003, p. 387). This inconsistency/divergence is the free energy, which has to be maintained at the lowest possible level to avoid surprise (Clark, 2013). The brain continuously generates prediction models based on noisy sensory inputs to represent future states of the body and the external world.

In our view, intentions work as prediction models with associated motivational value. Also a distal and abstract intention (“I want to become a psychologist”) is constructed and sharpened over time to match incoming external/sensory inputs. This process entails two main sub-level processes related to one’s own activity monitoring. The first entails the identification of affordances and opportunities for action (even in abstract terms) to understand whether and how the intention can be progressively accompanied to become an action. The second process consists in its progressive transformation in a more-and-more practical, concrete, and motor guide for action. In other words, the second process is the generation of the intentional cascade.

Let us consider an example. One person feels a motivational drive to study human behavior and to treat psychopathologies. For some reason, this has a positive emotional value for him and he considers these tasks as consistent with his own identity. So, he decides *he wants to be a psychologist*. This distal, abstract intention is not dependent on the here-and-now context. However, it does not start to guide behavior, nor it starts an intentional cascade, “out of nowhere”. The agent has to control whether or not there are, in his perception of the world, general opportunities to reach his own purpose: going to the university, following courses, augmenting his own knowledge, obtaining a psychology degree which would be accepted and recognized by the society he lives in. The distal intention has to match with thinkable opportunities in the world, this way starting to reduce the inconsistency between the representational content of the intention (“I want to become a psychologist”) and the current reality of the thinkable and perceivable world (“I am currently not a psychologist”).

Of course, at this level the free energy resulting from the inconsistency between the volitional representation and the actual state of the world is very high; moreover, it is probably impossible to represent it, because both the intention and the desired state exist just in abstract, imaginative terms. For this reason, while general opportunities for actions start to appear and to match with the intention, the intention itself has to be specified in here-and-now guides for action to adapt to more-and-more situated environmental affordances. This is “when” proximal intentions are generated, and have to be matched with the concrete opportunities for action existing in the current situation. Then, while proximal affordances are approached, motor affordances may appear informing how the physical movement should be performed. At this moment, the action can be initiated transforming proximal intentions in the best set of motor intentions for the situation. Doing this, the agent progressively fulfill his own distal intention and the entire intentional cascade, this way reducing the free energy resulting from the confrontation between the desired state (the representational content of the intention) and the actual state of the world.

**FIGURE 2:**
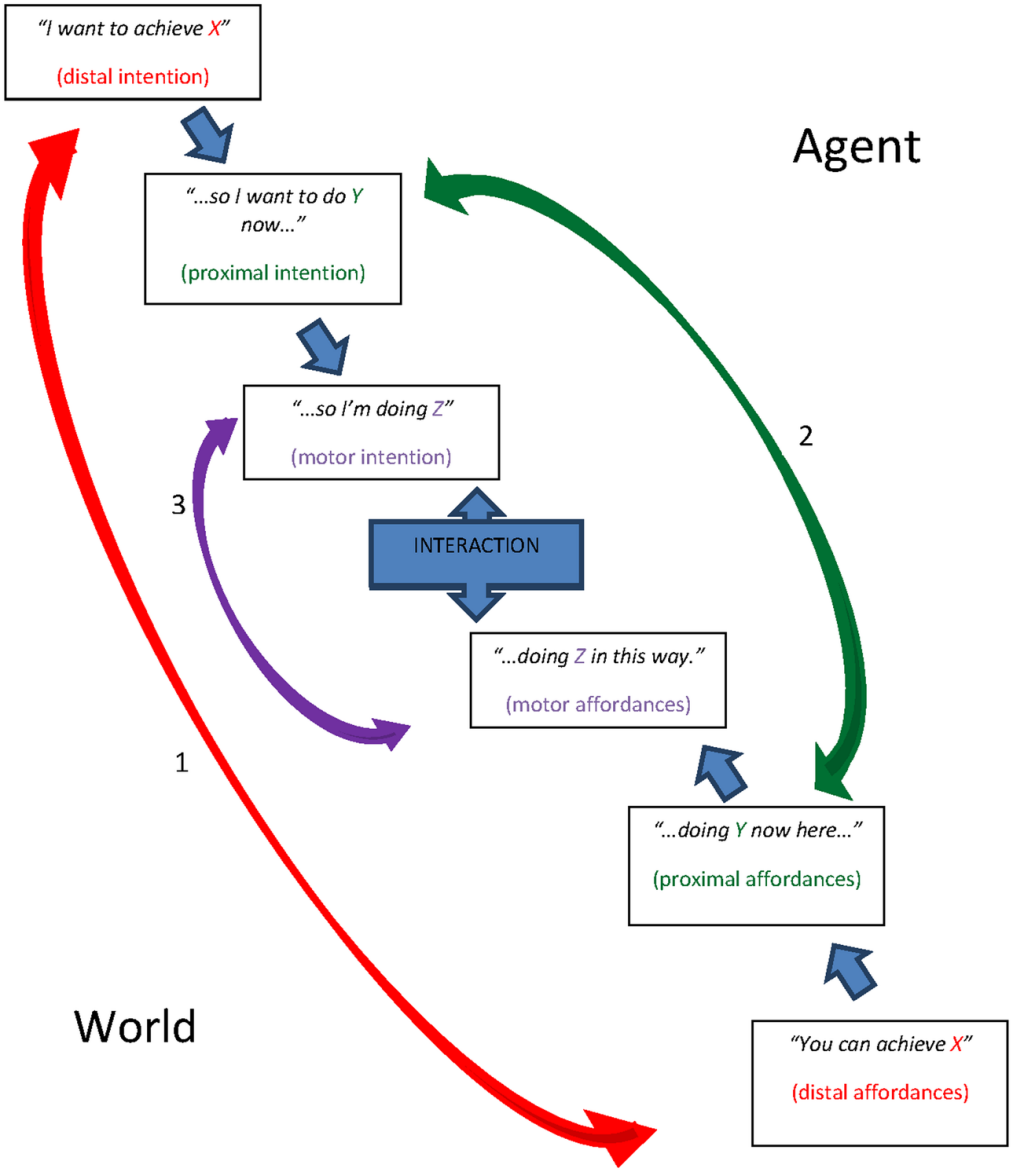
**The Model of Hierarchical Interlocking of Enacted Intentions, which is intended to show how any level of an intentional structure relates to a precise level of environmental affordances**.

**Figure 2** shows a theoretical model we originally proposed in the field of technology and human computer interaction (Triberti et al., n.d.; Triberti and Riva, 2015), originally labeled “Perfect Interaction Model” because it virtually represented the interaction in which every intentional level of the user perfectly-interlocks with the characteristics of a technology. Here, we present it as representing general intentional agency. In other words, the model represents the Hierarchical Interlocking of Enacted Intentions.

The model has six levels, three representing the human agent part (distal intention, proximal intention, motor intention) and the other three representing the world part (distal affordances, proximal affordances, motor affordances). The three arrows show how every intentional level interlocks with a precise level of the world’s opportunities for action.

Considering our example: the first arrow relates to the agent who wants to become a psychologist. He starts his own action plan in that the world actually presents the possibility to become a psychologist, containing possible courses of actions and cultural representations associated with this figure (distal affordances). Using Laurent’s (2003) terminology, the representational content of the distal intention resembles a “simulated affordance” in that it regards thinkable opportunities in the world to be achieved. The second arrow relates to the here-and-now intention to write an essay; it interlocks with the proximal affordances given by the set or “structure” (Garrett, 2010) of functions guaranteed by the computer the agent decides to use (a technology that allows one to write, cite, and save his own work); finally, arrow 3 represents the interlocking between the motor affordances of the computer (that is, the interface, or the physical representation of the structure of functions) and the motor intentions representing the movements to be performed (moving fingers *this way* to write a given letter).

In the field of Human Computer Interaction and User Experience, the present model is useful to identify the source of interaction failures at the level of intentional representation and/or technological features (Triberti et al., n.d.; Triberti and Riva, 2015) (for example: does the user ignore what to do, and so he doesn’t know how to structure action plans of use, or is the technology that doesn’t communicate well its own functions?). In this context, we argue that such a model may be useful to show how intentions relates to the external world already at the time when they are not transformed into physical action, through a process of continuous confrontation between representational content and the opportunities in the external world, devoted to progressive reduction of free energy.

As a conclusion, in accordance with both the theory of Intentions by Pacherie (2008) and the theory of Self by Damasio (2011), the presence theory from which we have started highlights that our own actions and intentions are enacted at a three-levels complexity; as motor behavior, based on proprioceptive information coming from our own bodies and their interaction with the physical properties of external objects; as proximal/contextual behavior, based on the perception of environmental functions and affordances; and as future behavior, based on the prefiguring/simulation of action plans. In this sense, any intentional hierarchy layer has to interlock with the respective environmental affordances, being them ready-to-hand physical properties (motor intentions), tools/obstacles actually present in the here-and-now environment (proximal intentions), or conceptualized action plans that are part of the society’s cultural background (distal-abstract intentions). The highest the success of the interlocking process at any level, the highest is the sense of being present in a situation, as the result of the impression of being able to transform intentions into actions and controlling one’s own agency in the world.

This contribution expands on the previous literature on the topic in two ways: on the one hand, it constitutes the first attempt to link the theory of presence to a modeling of intention enacting; on the other hand, it deepens the concept of intention highlighting its relationship with the world prior to its enacting.

Indeed, the described process happens on the background of sense of presence, that is, the sensation to be in a given situation emerging from the impression to be able to enact intentions. Sense of presence is not an automatic outcome of the “simple fact” that one find himself in a given place. On the contrary, we “know where we are” basing on our perceived possibility of being able to pursue our own objectives in distal, proximal, and motor terms.

Thanks to this fundamental process, we state the basis not only for the action plans related to the situated agency of motor behavior, but also of our own distal Self-projecting in future life.

## Conflict of Interest Statement

The authors declare that the research was conducted in the absence of any commercial or financial relationships that could be construed as a potential conflict of interest.
